# Evaluation of patients with COVID-19 diagnosis for chronic diseases

**DOI:** 10.1186/s12985-021-01524-0

**Published:** 2021-03-17

**Authors:** Murat Altuntas, Habip Yilmaz, Abdullah Emre Guner

**Affiliations:** 1 University of Health Sciences Bagcilar Training and Research Hospital, Istanbul, Turkey; 2 Provincial Health Directorate, Istanbul, Turkey

**Keywords:** Chronic disease, COVID-19, Diagnosis, Patient

## Abstract

**Aim:**

COVID-19 is one of the most consequential pandemic in world history. Chronic diseases, which are risk factors that increase the case fatality rates, have been the leading cause of death all over the world. This study was aimed at detecting coexisting chronic diseases in patients hospitalized with a diagnosis of COVID-19.

**Material and method:**

The study was carried out with data from 229 patients in an intensive care unit, from June 1st through June 30th. 2020. The inclusion criteria of the study was as follows: (1) having a COVID-19 diagnosis confirmed by PCR test; (2) being hospitalized in the relevant intensive care unit within the dates of the study; and (3) having their data accessible through the hospital automation system. Through literature; chronic diseases of the patients and their effects on the COVID-19 process were evaluated. Statistical analyzes were performed using the Statistical Package for Social Sciences (SPSS) version 24.0 (IBM Corp.; Armonk, NY, USA).

**Results:**

The average age of the patients studied were 61.4 years. While the average symptom duration was 8.2 days; total hospitalization period was 13.1 days. The average length of stay of patients (n = 75) who were sent to intensive care unit was 10.1. The most common chronic disease among patients was hypertension with 47.2%. This was followed by diabetes mellitus (32.8%) and heart disease (27.5%), respectively. In the population studied, cough (59.4%), fever (58.5%) and shortness of breath (45.9%) were found to be the most common symptoms. Leukopenia, impairments in liver and muscle enzymes, abnormal C-reactive protein, ferritin and d-dimer levels were the important biochemical tests.

**Conclusion:**

Particular attention should be paid to the elderly COVID-19 patients with chronic diseases, especially DM, HT and cancer.

## Introduction

December of 2019, a new severe acute respiratory syndrome, COVID-19, was reported in Wuhan, China. The virus causing this airborne disease was determined to be coronavirus-2 (SARS-CoV-2) [1]. COVID-19 is the largest pandemic in the world after H1N1 influenza epidemic in 1918 [2]. Based on the current studies the clinical course of the disease varies from mild upper respiratory tract infection findings to severe viral pneumonia accompanied by loss of taste and smell and respiratory failure [3].

Although the virus infects individuals of all ages; it is known that people at an older age and with concomitant chronic diseases have more severe symptoms. Studies show that among the the increasing number of cases mostly affected populations are people with previously known chronic diseases [4]. Risk factors associated with serious disease and mortality are advanced age, cardiovascular disease (CVD), diabetes mellitus (DM), hypertension (HT), chronic lung disease, cancers, chronic kidney disease (CKD), use of immunosuppressive or biological agents, obesity, and smoking [5]. These diseases, which are risk factors that increase the case fatality rates, have been the leading cause of death in all developed or developing countries around the world [6].

Chronic diseases affect mortality with different mechanisms during COVID-19. It is known that there is an increase in troponin level associated with disease severity and mortality during the course of the disease. Severe viral infections causing systemic inflammatory syndrome increase the risk of plaque, rupture and thrombus formation, and thus result in cardiovascular events [7]. In the course of COVID-19, microangiopathic changes occurring in the respiratory tract of diabetic patients reduce gas exchange and lung compliance and cause a significant decrease in forced vital capacity (FVC) and forced expiratory volume in 1.second (FEV1) [8]. Hypertension is another important disease that need to be placed attention in Covid patients. SARS-CoV-2 enters target cells by binding to angiotensin converting enzyme 2 (ACE2) expressed on epithelial cells of lung, kidney, blood vessels. ACE2 expression increases in patients with HT and DM, who are treated with ACE inhibitors or receptor blockers [9].

It is known that the rates of chronic diseases are also high in regions with high mortality rates such as China, Europe, and the United States [10]. Identifying additional risk factors associated with COVID-19 patients will also affect the survival of individuals in this group. In this study, it is aimed to detect coexisting diseases in patients hospitalized with the diagnosis of COVID-19.

## Material and method

The study was carried out in Health Sciences University Bağcılar Training and Research Hospital Adult Intensive Care Unit, from June 1st through June 30th. 2020. It is a prospective, cross-sectional study. Study participants were selected based on the following criteria; (1) to have a diagnosis confirmed by Polymerase Chain Reaction (PCR) test, (2) to be hospitalized in the relevant intensive care unit on the date of the study and (3) to have data accessible through the hospital automation system. A total of 229 patients who have these criteria were included in the study.

Study was approved by the Ethics Committee of Istanbul Göztepe Training and Research Hospital, Turkey with the decision number 2020/0243. In addition; TR Ministry of Health Scientific Research Platform on COVID-19 has also obtained a work permit with the date 04.05.2020 and number T190535.

Statistical analysis was performed by IBM SPSS Statistics 24 program. Descriptive data are presented by giving percentage distributions and mean ± standard deviation. T-test was used for measurement data in independent groups and Pearson chi-square test was used for census data in examining causality relationships. Considering the 95% confidence interval and 5% margin of error in the analysis, *p* < 0.05 was accepted as a significance level.

## Results

Total 75 patients (32.8%) of the study group were female and 154 (67.2%) were male. It was seen that the average age of the patients was 61.4 years old. While the average symptom duration was 8.2 days; total hospitalization period was 13.1 days. The average length of stay of 75 patients who were sent to intensive care unit was determined as 10.1.

**Fig. 1:**
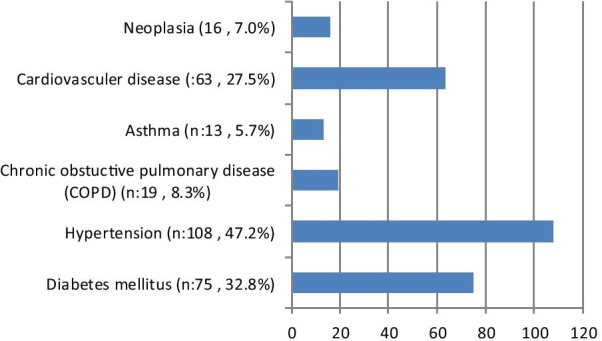
Distribution of the patients according to their chronic diseases. * Those with more than one registered disease at the same time in the automation system were included in all groups separately

The most common chronic disease among patients was hypertension with 47.2%. This was followed by diabetes mellitus (32.8%) and heart disease (27.5%), respectively. The distribution of patients according to their chronic diseases was given in Fig. 1.

Distribution of the COVID-19 inpatients according to their symptoms and the treatments they received; was presented in Table 1.

**Table 1:** Distribution of the patients according to their symptoms and medications

	N^a^	%
Fever	134	58.5
Cough	136	59.4
Shortness of breath	105	45.9
Runny nose	5	2.2
Clinic		
Mild	1	0.4
Moderate	116	50.7
Bad	21	9.2
Terrible	91	39.7
Intensive care admission	93	40.6
Oseltamivir treatment	154	67.2
Favipiravir treatment	229	100.0
Combined therapy	101	44.1
Vitamin C	117	51.1
Cortikosteroid	34	14.8
Anticoagulant therapy	192	83.8
Mechanical ventilation	67	29.3
Result (n = 227)		
Exitus	55	24.0
Hospitalization continues	135	59.5
Admission to intensive care	32	14.0
Hospitalization in another service	1	0.4

^a^Those with more than one registered disease at the same time in the automation system were included in all groups separately

According to the symptom distribution of the patients; it was observed that cough, fever and shortness of breath were observed with close frequency. Among the symptoms of fever, cough, shortness of breath and runny nose, the least common symptom was runny nose with 2.2%. Considering the distribution of the disease according to its clinical severity; it was seen that the most common clinical presentation was "moderate" (50.7%). While all patients (100%) recieved the favipiravir treatment, only 14.8% of the patients recieved corticosteroid treatment. When the treatment results of the patients examined, it is observed that 59.5% were still hospitalized during the period data were collected, and 24% died. Biochemical values of patients during initial hospitalization ​​are presented in Table 2.

**Table 2:** Summary of biochemical investigations in hospitalized COVID-19 patients

	N	Minimum	Maximum	Average	Std. deviation
Leukocyte	228	830.0	59,770.0	8036.7	5571.9
BUN	227	13.0	244.1	44.6	32.6
Creatinin	210	.3	10.3	1.3	1.2
Aspartat aminotransferaz (AST)	229	9.0	2028.0	47.5	134.9
Alanin aminotransferaz (ALT)	229	6.0	561.0	38.6	45.2
C-reaktif protein (CRP)	219	.01	318.4	87.1	73.4
Procalcitonin	107	.01	20.0	1.4	3.5
Ferritin	154	13.0	36,872.5	925.6	3107.0
D-dimer	209	0.01	27.7	1.2	2.5

After the analysis of post descriptive data, causality comparisons were performed. Causality comparisons were conducted based on gender and chronic diseases.

### Causality comparisons by gender

When the distribution of chronic diseases by gender was examined; DM, HT and asthma were significantly higher in females than males (respectively; χ^2^ = 6.407, *p*: 0.016; χ^2^ = 7.370, *p*: 0.007; Fisher's *p*: 0.011). Chronic obstructive pulmonary disease (COPD) is significantly higher in men than women (χ^2^ = 4.647, *p*: 0.031).

When the distribution by gender is examined in terms of the severity of the clinical picture and prognosis; there was no significant difference between the two sexes (χ^2^ = 2.396, *p*: 0.494; χ^2^ = 3.242, *p*: 0.198, respectively). When the results of the patient treatment were examined according to gender, no significant difference was found (χ^2^ = 4.034, *p*: 0.401). In addition, when disease symptoms were examined by gender, no significant difference was found in terms of fever, cough, shortness of breath and nasal discharge (χ^2^ = 1.950, *p*: 0.163; χ^2^ = 0.195, *p*: 0.659; χ^2^ = 0.154, *p*: 0.695; Fisher's *p*: 0.695, respectively).

T test analysis of age distribution by gender; revealed that women are significantly older than men (t test *p*: 0.007). Hospitalization C-reactive protein (CRP) value was found to be significantly higher in men than women (t test: *p*: 0.001).

### Causality comparisons according to chronic diseases

When the symptoms of fever, cough, shortness of breath and runny nose are examined according to chronic diseases; there was no difference in the presence of DM, COPD, neoplasia. Those with HT are found to be significantly higher than those without fever (χ^2^ = 4.850, *p*: 0.028). Patients with asthma were found significantly more likely to present shortness of breath than the others (χ^2^ = 8.341, *p*: 0.004). The patients with heart disease were significantly higher in presenting cough and shortness of breath than those without the disease (χ^2^ = 4.992, *p*: 0.025; χ^2^ = 4.463, *p*: 0.035, respectively).

Considering the clinical picture of patients with DM, the rate of bad and severe cases were found significantly higher than those without DM(χ^2^ = 10.466, *p*: 0.015). Similarly, the rates of admission to intensive care unit and exitus were higher in this patient group (χ^2^ = 7.584, *p*: 0.006; χ^2^ = 10.367, *p*: 0.035).

There were no significant differences found in clinical picture and outcome of patients with HT. The rates of admission to intensive care unit were higher in this patient group than those who don’t have HT (χ^2^ = 6.069, *p*: 0.014).

No significant difference were found between those with and without COPD and asthma in terms of clinical picture, prognosis, admission to intensive care and outcome compared to those without COPD.

When compared with those without heart disease, those with heart disease admitted to intensive care were higher (for ICU admission χ^2^ = 6.429, *p*: 0.011).

When patients with neoplasia and those without neoplasia are compared; it was seen that the clinical picture was significantly worse in patients with neoplasia than those without neoplasia (χ^2^ = 16.721, *p*: 0.001). It was also found that the rate of exitus in patients with neoplasia was significantly higher than in those without neoplasia (χ^2^ = 16.147, *p*: 0.003).

### Cross comparisons of chronic diseases and biochemical tests

While blood urea nitrogen (BUN) and CRP were found to be significantly higher in DM patients than those without DM, the duration of symptoms was shorter (t test: *p*: 0.0001, *p*: 0.001, *p*: 0.042, *p*: 0.005, respectively).

While BUN, procalcitonin and d-dimer were found to be significantly higher in patients with HT than those without HT, the symptom duration was shorter (t test: *p*: 0.0001, *p*: 0.001, *p*: 0.0001, *p*: 0.007, *p*: 0.008, respectively).

Age, CRP, and total length of stay were significantly higher in patients with COPD than those without (t test: *p*: 0.001, *p*: 0.009, *p*: 0.007, respectively). Symptoms and duration of intensive care were found to be lower in patients with asthma compared to those without intensive care (t test: *p*: 0.011, *p*: 0.036, respectively). There was no significant difference for the others.

Age, BUN, creatinine and non-d-dimer were significantly higher in those with heart disease than those without. The symptom duration was lower (t test: *p*: 0.0001, *p*: 0.0001, *p*: 0.035, *p*: 0.004, *p*: 0.015, respectively).

## Discussion

In the studies conducted in different countries during the first period of the global epidemia, it was found that most of the patients were male and advanced age [11–13]. In one of the first studies on this field conducted in Wuhan, it was reported that the patients were mostly composed of elderly women and the most important diseases associated were hypertension, diabetes, heart diseases and COPD [14]. In this study similar results were observed in age and gender (male ratio 67.2%, mean age 61.4 ± 15.9).

Hypertension was found to be the most common chronic disease associated with patients diagnosed with COVID-19 in the study (47.2%). High risk of serious disease in hypertensive patients was thought to be related; with the use of angiotensin converting enzyme 2 (ACE2) as the viral entry receptor in lung cells by the virus, with the high prevalence of hypertension in the population, and the frequency of use of renin-angiotensin system (RAS) blockers [14]. In the study, although fever in these patients is statistically significantly higher than other symptoms, the mechanism of this is not fully known. In different studies, it is emphasized that hypertension is a common comorbidity for COVID-19 infection, which significantly affects mortality and disease severity [11, 12, 15, 16].

The frequency of diabetes mellitus in the study was found to be 32.8%. With this rate, diabetes is the second most common disorder among the patients who participated in the study. For diabetes mellitus, Huang et al. reported as the main comorbidity disease with a frequency of 20% [11]. Its’ frequency was found around 10% in different studies [17].

Cardiovascular diseases were found to be the third most common chronic disease in the study group (27.5%). Case series with additional cardiovascular disease have also been reported in the literature [18, 19].

Although the disease affects the respiratory tract; Huang et al. [11] and Wang et al. [18] found the prevalence of accompanying COPD to be 2% and 2.9%, respectively. Similarly, Chen et al. [19] found associated respiratory system diseases 1% in their study. In the study, COPD was seen at a very high rate with 8.3% and asthma 5.7%. These high rates found in the study have been associated with the high frequency of respiratory system diseases. In addition, rare, bronchiectasis, interstitial lung disease and tobacco use have been reported as coexisting risk factors alone [12, 13, 20, 21]. Moreover; in patients with asthma, the statistically significant excess of shortness of breath was associated with the clinical course of the disease.

Advanced DM, HT, neoplasia and heart disease patients with COVID-19 required significantly more intensive care. There was no statisticly significant relationship found between the need for intensive care and the severity of the disease with COPD. This finding might be contributed to the size of the population studied, and we recommend caution in interpreting this finding. We recommend more studies conducted to detremine the relationship between the need for intensive care and the severity of the disease with COPD.

Neurological diseases are the disease group that cause the highest health burden especially in the elderly population [22]. Despite this, no neurological disease was found in the records of patients diagnosed with COVID-19 in the study. Considering that neurological diseases require long-term follow-up and treatment; even if there is no COVID-19 diagnosis, special attention should be paid to the follow-up of these patients.

In present study, no findings regarding with digestive system disorders and COVID-19 were found. However; it has been reported that some patients with COVID-19 show digestive symptoms such as diarrhea, vomiting and abdominal pain, and the average liver enzyme levels of these patients were also high [23]. Although it has been stated that inflammatory bowel diseases such as Crohn's and colitis ulcerosa may be diseases in patients with COVID-19 that contribute as genetic predisposition and environmental factors [24], no findings were found in this study. This finding might be attributed to the study group in the intensive care unit, and this finding need to be interpreted cautiously.

Cough (59.4%), fever (58.5%) and shortness of breath (45.9%) were found to be the most common symptoms in this study. This situation was parallel to the literature [3, 19, 21].

In the conducted studies, leukopenia is the most common laboratory finding; rarely, thrombocytopenia is observed [3, 12, 25]. In the literature, impairments have also been reported in liver and muscle enzymes. Abnormal elevations can also be observed in C-reactive protein, ferritin and d-dimer levels [16, 17].

## Conclusion

It would be appropriate to evaluate carefully to COVID-19 patients, especially for the current chronic diseases. Particular attention should be paid to the elderly COVID-19 patients with chronic diseases, especially DM, HT and cancer.

## Data Availability

Not applicable.

## Abbreviations

ACE-2: Angiotensin converting enzyme-2
BUN: Blood urea nitrogen
CKD: Chronic kidney disease
COPD: Chronic obstructive pulmonary disease
CRP: C-Reactive protein
COVID-19: Severe acute respiratory syndrome 2, SARS-2
CVD: Cardiovascular disease
DM: Diabetes mellitus
FEV-1: Forced expiratory volume in 1 s
FVC: Forced vital capacity
HT: Hypertension
ICU: Intensive care unit
PCR: Polymerase chain reaction
RAS: Renin angioensin system
SPSS: Statistical package for social sciences
